# Pan-cancer characterization of cell-free immune-related miRNA identified as a robust biomarker for cancer diagnosis

**DOI:** 10.1186/s12943-023-01915-7

**Published:** 2024-02-12

**Authors:** Peng Wu, Chaoqi Zhang, Xiaoya Tang, Dongyu Li, Guochao Zhang, Xiaohui Zi, Jingjing Liu, Enzhi Yin, Jiapeng Zhao, Pan Wang, Le Wang, Ruirui Li, Yue Wu, Nan Sun, Jie He

**Affiliations:** 1 Department of Thoracic Surgery, National Clinical Research Center for Cancer/Cancer Hospital, National Cancer Center, Chinese Academy of Medical Sciences, Peking Union Medical College, Beijing, 100021 China; 2 4+4 Medical Doctor Program, Chinese Academy of Medical Sciences and Peking Union Medical College, Beijing, 100021 China; 3 Department of Otolaryngology-Head and Neck Surgery, The First Affiliated Hospital of Zhengzhou University, Zhengzhou, 450052 China; 4 Department of Pathology, National Clinical Research Center for Cancer/Cancer Hospital, National Cancer Center, Chinese Academy of Medical Sciences and Peking Union Medical College, Beijing, 100021 China; 5 Department of Clinical Laboratory, National Clinical Research Center for Cancer/Cancer Hospital, National Cancer Center, Chinese Academy of Medical Sciences, Peking Union Medical College, Beijing, 100021 China

**Keywords:** Cell-free immune-related miRNAs, Pan-cancer analysis, Machine learning algorithms, Early detection of cancers

## Abstract

Minimally invasive testing is essential for early cancer detection, impacting patient survival rates significantly. Our study aimed to establish a pioneering cell-free immune-related miRNAs (cf-IRmiRNAs) signature for early cancer detection. We analyzed circulating miRNA profiles from 15,832 participants, including individuals with 13 types of cancer and control. The data was randomly divided into training, validation, and test sets (7:2:1), with an additional external test set of 684 participants. In the discovery phase, we identified 100 differentially expressed cf-IRmiRNAs between the malignant and non-malignant, retaining 39 using the least absolute shrinkage and selection operator (LASSO) method. Five machine learning algorithms were adopted to construct cf-IRmiRNAs signature, and the diagnostic classifies based on XGBoost algorithm showed the excellent performance for cancer detection in the validation set (AUC: 0.984, CI: 0.980–0.989), determined through 5-fold cross-validation and grid search. Further evaluation in the test and external test sets confirmed the reliability and efficacy of the classifier (AUC: 0.980 to 1.000). The classifier successfully detected early-stage cancers, particularly lung, prostate, and gastric cancers. It also distinguished between benign and malignant tumors. This study represents the largest and most comprehensive pan-cancer analysis on cf-IRmiRNAs, offering a promising non-invasive diagnostic biomarker for early cancer detection and potential impact on clinical practice.

**Supplementary Information:**

The online version contains supplementary material available at 10.1186/s12943-023-01915-7.

## Main text

Cancer is recognized as a severe public health problem, with increasing morbidity and mortality worldwide [1]. Despite therapeutic advancements, the prognosis of cancers remains grim. Early detection is crucial for improved outcomes, but current biomarkers and techniques are inadequate for widespread screening [2, 3]. Hence, finding practical, minimally invasive approaches for early cancer detection are of great significance. Cell-free miRNAs (cf-miRNAs) offer promise as liquid biopsy markers due to their stability and abundance [4]. Considering inflammatory reactions and biomarkers may precede cancer diagnosis by years, and the immunosuppressive microenvironment resulting from chronic inflammation can contribute to the development and activation of cancer. Over the past decade, various miRNA-based signatures have been developed to diagnose certain cancer types [5–9], however, the limited sample size and incomplete model construction methods hinders their clinical utility. Also, few study focused on the diagnostic performance of immune-related miRNAs. Therefore, we attempted to investigate cell-free immune-related miRNA profiles (cf-IRmiRNAs) between malignancies and non-malignancies, exploring their diagnostic utility.

**Fig. 1:**
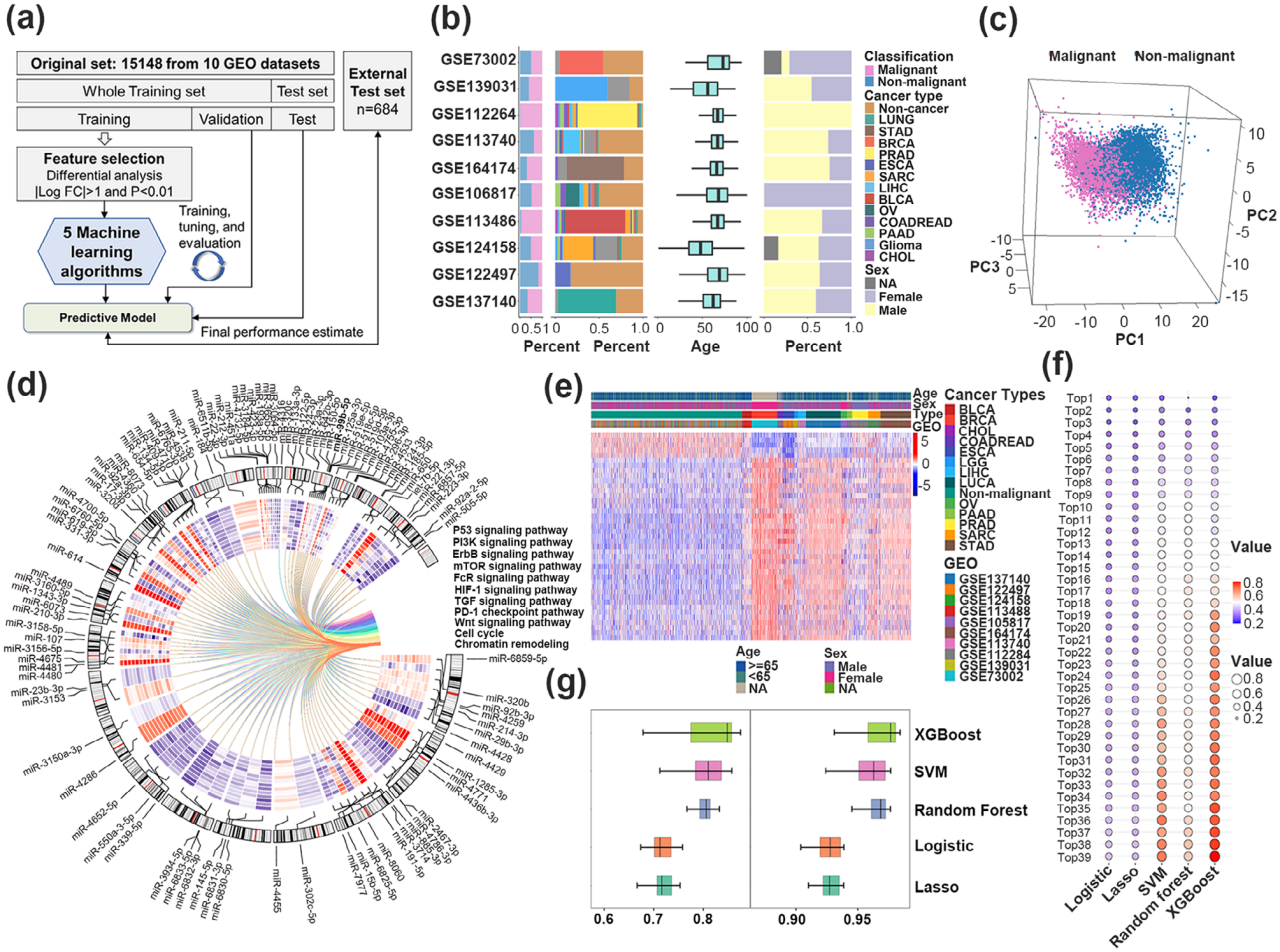
The profile of cell free immune-related circulating miRNAs (cf-miRNAs) between malignancies and non-malignancies. **(a)** Workflow of the study. **(b)** Distribution of the number of samples, histological type, age, and sex in 10 GEO datasets. Clinical samples include lung cancer (LUCA, n = 1606), esophageal cancer (ESCA, n = 601), gastric cancer (STAD, n = 1447), Liver hepatocellular carcinoma (LIHC, n = 466), colorectal cancer (COADREAD, n = 272), breast cancer (BRCA, n = 1285), prostate cancer (PRAD, n = 809), pancreatic cancer (PAAD, n = 227), ovarian cancer (OV, n = 327), bladder Cancer (BLCA, n = 392), sarcoma (SARC, n = 591), glioma (n = 212), biliary tract cancer (CHOL, n = 81), and 7516 non-cancer individuals (health, other diseases, and benign tumors). **(c)** Principal component analysis (PCA) analysis of malignancies and non-malignancies based on differentially expressed miRNAs. **(d)** Circos plot showing the differentially expressed miRNAs immune pathway among malignancies. The inner heatmap showed the expression of miRNAs across cancer types. **(e)** Heatmap showed a significant difference between malignancies and non-malignancies based on 39 cf-miRNAs in the validation set. **(f)** Youden index of each classifier in the validation set. The X-axis is five types of machine learning algorithms and the Y-axis is Youden value. The redder means a higher value. **(g)** Youden index (left) and area under curve (AUC) (right) performance for each classifier

Pan-Cancer study analyzed 15,832 samples from 13 cancer types and non-malignant individuals with non-coding RNA profiles, including lung cancer, esophageal cancer, gastric cancer, liver cancer, colorectal cancer, breast cancer, prostate cancer, pancreatic cancer, ovarian cancer, bladder cancer, biliary tract caner, sarcoma, and glioma. The workflow and the specific clinical information of these samples are provided in Fig. 1a-b, Fig. S1 and Table S1. A catalog of 1,256 immune miRNAs was curated (Table S2), and probes with a flag value above 3 in more than half of the samples were defined as abundant serum miRNAs (515 miRNAs). The panorama of the candidates cf-IRmiRNAs in malignancies and non-malignant samples was evaluated through principal component analysis (PCA), which revealed a dramatically different distribution pattern (Fig. 1c).

To identify reliable candidate cf-IRmiRNAs that showed differential representation between malignant and non-malignant controls, differential analysis was performed in the training set, and we identified 100 differentially expressed cf-IRmiRNAs (|logFC| > 1, *P* value < 0.01) (Table S3). Then, we conducted a detailed presentation and pathway annotation of those differentially expressed miRNAs, and the results of the selected cf-IRmiRNAs were largely correlated with immune pathways, including the PI3K signaling pathway, PD-1 signaling pathway in cancer, and the Wnt signaling pathway (Fig. 1d and Table S4). Using Lasso regression (λ = 0.008), we retained 39 miRNAs, and hierarchical clustering analysis showed distinct expression patterns between malignant and non-malignant samples based on that (Fig. 1e).

The diagnostic performance of a single candidate miRNA was explored in the training set. Individual miRNAs showed promising diagnostic utility, with hsa-miR-17-3p performing the best (Area under curve (AUC): 0.878, sensitivity: 0.823, specificity: 0.799). (Fig. S2a and Table S5). Notably, the identified miRNAs also exhibited remarkable diagnostic performance in lung, esophageal, gastric, and breast cancers (AUC > 0.8) (Fig. S2b-c and Table S6), which consisted of previous studies [10–12].

**Fig. 2:**
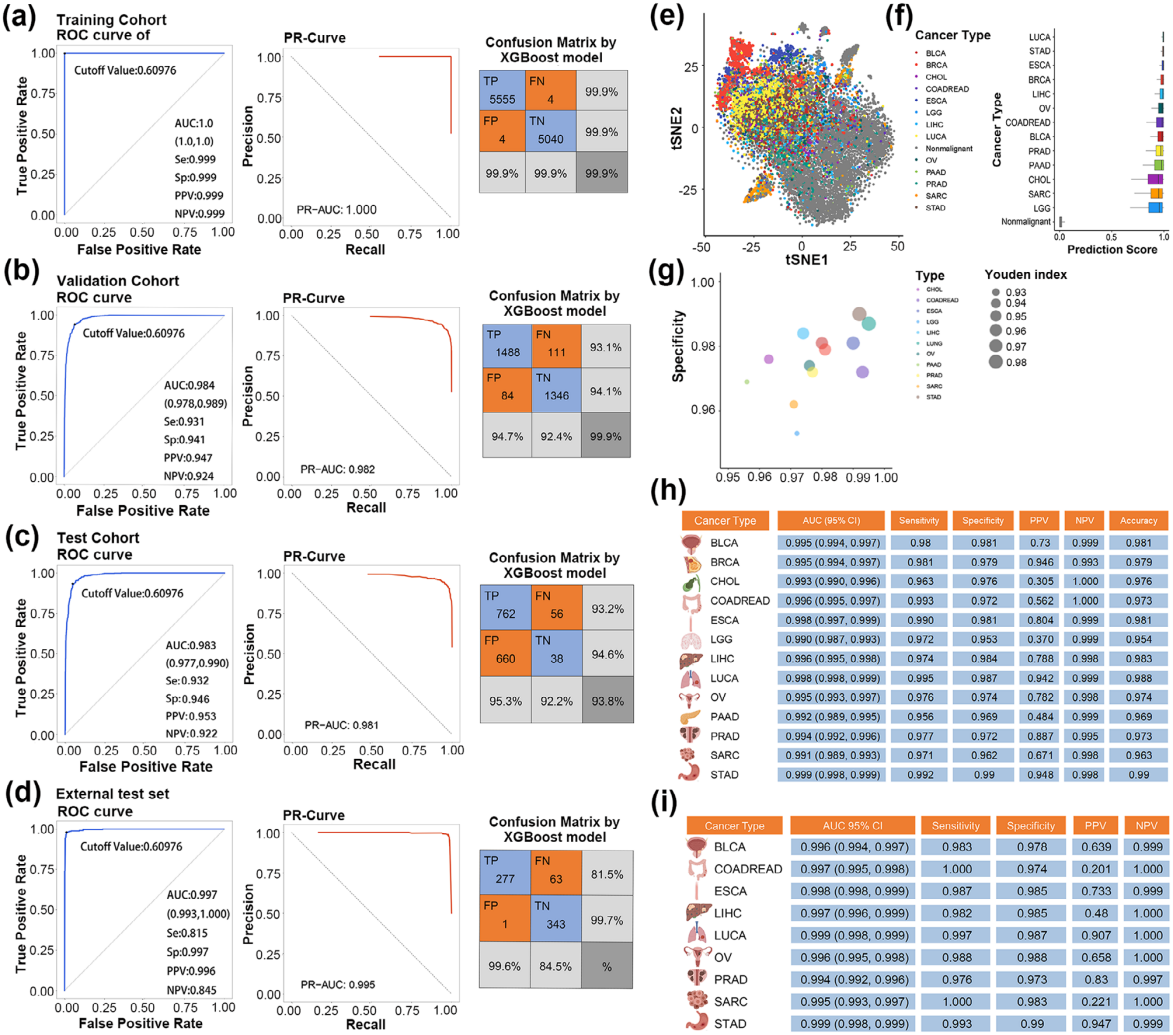
The diagnostic utility of cell-free immune-related miRNAs (cf-IRmiRNA) signature in detecting cancers. **a-d.** Receiver operating characteristic curve (ROC), PR curve, and confusion matrix for cf-IRmiRNA signature for cancer diagnosis in the train, validation, test, and external test set. **e.** t-SNE analysis of the samples from the whole cohort. **f.** The diagnostic index of cf-IRmiRNAs signature in participants. **g.** Scatter plot showing the sensitivity and specificity of the diagnostic index in 13 types of cancer. Size indicates the value of Youden index. **h.** Summary of AUC, sensitivity, specificity, positive predictive value (PPV), and negative predictive value (NPV) of the cf-IRmiRNAs signature in distinguishing each cancer type from non-cancer controls. **i.** Summary of the early diagnostic utility of the identified signature among each cancer type from non-cancer controls

In the subsequent stage, we employed five widely used algorithms, including Logistic regression, Lasso regression, Support Vector Machine (SVM), Random Forest, and eXtreme Gradient Boosting (XGBoost) to integrate the 39 selected cf-IRmiRNAs and construct diagnostic classifiers. The XGBoost algorithm with 39 miRNA outperformed others with an AUC of 0.983 in discriminating cancers and controls (sensitivity: 0.931, specificity: 0.945) in the validation set (Logistic AUC: 0.939, Lasso AUC: 0.938, Random Forest AUC: 0.976, and SVM AUC: 0.976) (Fig. 1f-g and Table S7). As expected, the classifier demonstrated superior performance compared to single miRNAs (Fig. S3a-b). Through parameter tuning and 5-fold cross-validation, the 39-cf-IRmiRNAs signature achieved an improved performance with an AUC of 0.984 (95% CI: 0.980, 0.989), sensitivity of 0.931 (95%CI: 0.922,0.940), and specificity of 0.941 (95%CI: 0.933,0.950) in the validation set (Fig. 2a-b and Table S8). The classifier also achieved high performance with an AUC of 0.983 (95% CI: 0.977, 0.990), sensitivity of 0.932 (0.932, 95% CI: 0.919, 0.944), and specificity of 0.946 (0.946, 95% CI: 0.934, 0.957) in the test set (Fig. 1c). Further validation in external test sets (AUC: 0.997, 95% CI: 0.993-1.000; Sensitivity: 0.815, 95% CI: 0.786–0.844; Specificity: 0.997, 95% CI: 0.993,1.000) and the entire cohort confirmed the stability and superiority of our signature (Fig. 1d and Fig. S4a-c). Except that, the hsa-miR-17-3p act as an individual diagnostic biomarker, and was well validated in the validation, test, and external test sets with an AUC of 0.887 (0.875–0.899), 0.888 (0.871–0.905), and 0.776 (0.741–0.811), respectively (Table S9).

To evaluate the ability of the cf-IRmiRNA signature in distinguishing cancer types, we analyzed the miRNA profiles in each cancer type individually with non-malignant samples. T-distributed stochastic neighbor embedding (TSNE) was used to visualize the differences between cancer types based on differentially expressed cf-IRmiRNAs in a lower-dimensional space (Fig. 2e). The diagnostic index, calculated with the cf-IRmiRNAs signature showed higher scores in malignant samples than that of the non-malignant ones (Fig. 2f). Moreover, the diagnostic index showed a high discriminant performance in each cancer type, especially in lung cancer (AUC: 0.998, 95% CI: 0.998–0.999, sensitivity: 0.995, sp: 0.987, positive predictive value (PPV): 0.942, negative predictive value (NPV): 0.999), ESCA (AUC: 0.998, 95% CI: 0.997, 0.999, sensitivity: 0.990, specificity: 0.981, PPV: 0.804, NPV: 0.999), and STAD (AUC: 0.999, 95% CI: 0.998–0.999, sensitivity: 0.992, specificity: 0.990, PPV: 0.984, NPV:0.998) (Fig. 2g and h). Although the positive predictive value (PPV) of cf-IRmiRNAs signature in certain types of cancer was a little weakened, the classifier showed remarkably high negative predictive value (NPV). This meant that the classifier is more applicable for cancer screening, which can maximize the detection of positive cases and reduce delayed cancer diagnosis. Notably, the classifier still exhibited outstanding performance in early-stage cancer detection, especially in lung and gastric carcinoma, with an AUC of 0.990 (Fig. 2i).

Additionally, we verified the potential utility of cf-IRmiRNA signature for distinguishing between benign and malignant lesions within the corresponding organs or tissues. In the same organs or tissues, the diagnostic index of malignancies was significantly higher than that of the benign lesions (Fig. S5a, c, e, g, and i). ROC analysis confirmed its effectiveness in differential diagnosis across various tissues, including mesenchymal tissues, breast, liver, prostate, and ovary, with AUC achieved 0.955, 0.904, 0.999, 0.994, and 0.928, respectively (Fig. S5b, d, f, h, and j).

In this study, we revealed the value of cf-IRmiRNAs for cancer detection based on the largest sample-sized cohorts and highlighted the great potential of cf-IRmiRNAs panels for accurate noninvasive detection of early-stage malignancies with high accuracy. Considering the retrospective design of the study, further validation in large-scale prospective and multicentral trials is needed.

### Electronic supplementary material

Below is the link to the electronic supplementary material.

Supplementary Material 1

Supplementary Material 2

## Data Availability

The datasets used in this study are publicly available. All other relevant data and codes are available upon request.

## Abbreviations

cf-IRmiRNAs: cell-free immune-related miRNAs
AUC: Area under the curve
PCA: Principal component analysis
RF: Random Forest
LASSO: Least Absolute Shrinkage and Selection Operator
SVM: Support Vector Machine
XGBoost: eXtreme Gradient Boosting
TSNE: T-distributed stochastic neighbor embedding
