# Supplementary material for: Pan-cancer characterization of cell-free immune-related miRNA identified as a robust biomarker for cancer diagnosis
Source: Mol Cancer. 2024 Feb 12;23:31. doi: 10.1186/s12943-023-01915-7 (PMC10860228; doi:10.1186/s12943-023-01915-7)
Supplement: Supplementary file 2 — Supplementary Material 2 [file 12943_2023_1915_MOESM2_ESM.docx]

**Supplementary Files for**

**Pan-cancer characterization of cell-free immune-related miRNA identified as a robust** **biomarker for cancer diagnosis**

Peng Wu^1#^, Chaoqi Zhang^1#^, Xiaoya Tang^1#^, Dongyu Li^1,2^, Guochao Zhang^1^, Xiaohui Zi^1^, Jingjing Liu^1^, Enzhi Yin^1^, Jiapeng Zhao^2^, Pan Wang^1^, Le Wang^3^, Ruirui Li^4^, Yue Wu^5^, Nan Sun^1*^, Jie He^1*^

1. Department of Thoracic Surgery, National Cancer Center/National Clinical Research Center for Cancer/Cancer Hospital, Chinese Academy of Medical Sciences and Peking Union Medical College, Beijing, 100021, China
2. 4+4 Medical Doctor Program, Chinese Academy of Medical Sciences and Peking Union Medical College, Beijing, 100021, China
3. Department of Pathology, National Cancer Center/ National Clinical Research Center for Cancer/Cancer Hospital, Chinese Academy of Medical Sciences and Peking Union Medical College, Beijing, 100021, China
4. Department of Otolaryngology-Head and Neck Surgery, The First Affiliated Hospital of Zhengzhou University, Zhengzhou 450052, China.
5. Department of Clinical Laboratory, National Cancer Center/National Clinical Research Center for Cancer/Cancer Hospital, Chinese Academy of Medical Sciences and Peking Union Medical College, Beijing 100021, China.

Running title: cell-free immune-related miRNA in cancer detection

#These authors contributed equally to this work.

*Correspondence to: Jie He (prof.jiehe@gmail.com) and Nan Sun (sunnan@cicams.ac.cn)

**Method and materials**

**Study design and population**

We conducted a retrospective study with the largest sample size to date to collect and analyze the miRNA expression profiles of malignant and non-malignant samples. Our goal was to develop a new tool for early detection of cancers. Serum miRNA array data and corresponding clinical information were obtained from ten publicly available datasets from the Gene Expression Omnibus (GEO), including GSE137140[1], GSE122497[2] GSE124158[3], GSE113486[4], GSE106817[5], GSE164174[6], GSE113740[7], GSE112264[8], GSE139031[9], and GSE73002[10].

The inclusion criteria were as follows: a) histologically diagnosed, and b) no prior or ongoing cancer treatment before serum collection. The exclusion criteria included a) duplicate samples and b) samples with different clinical information but corresponding to the same individuals. After eliminating duplicate and inconsistent clinical information samples across datasets, a total of 8,316 cancer patients and 7,516 non-malignant controls were included in our study. The serum samples contained 13 types of cancer, including lung cancer (LUCA), esophageal cancer (ESCA), gastric cancer (STAD), liver cancer (LIHC), colorectal cancer (COADREAD), breast cancer (BRCA), prostate cancer (PRAD), pancreatic cancer (PAAD), ovarian cancer (OC), bladder cancer (BLCA), sarcoma (BLCA), glioma, and biliary tract cancer (CHOL). The non-cancer control group included not only healthy volunteers but also patients with benign lesions in the corresponding organs or tissues.

**Collection of cell-free miRNAs**

All datasets were based on a standardized microarray platform of 3D-Gene Human miRNA V21_1.0.0 (GPL21263 and GPL18941). In brief, miRNAs were considered to be present if their microarray signal was greater than [mean + 2 × standard deviation] of the negative control signals, from which the top and bottom 5%, ranked by signal intensity, were removed. When the miRNA expression signal was negative or undetectable after background subtraction, it was replaced by 0.1 on a scale of the base 2 logarithm. Details of the miRNA sequencing processing procedures was described in previous studies. All the datasets were merged, and batch effects were mitigated using the ComBat package.

**Identification of immune-related miRNA**

AmiGO 2 is a web-based set of tools for searching and browsing gene ontology databases[11]. 3,127 Human immune-related genes (IRGs) were downloaded from the AmiGO 2 Web portal (https://amigo-20231025.geneontology.org/amigo). Subsequently, we identified 1,256 immune-related miRNAs whose target genes were consistently expressed in three predictive datasets (TargetScan, miRDB, and miRTarBase)[12-14]. These datasets serve as comprehensive atlases of predicted and validated targets of biologically relevant miRNA-mRNA interaction networks. The detailed list of immune-related miRNAs is provided in Table S2. Additionally, we screened 515 miRNAs that were highly stable and expressed, with log2-transformed values above three in more than half of the samples, for further analysis.

**Pathway enrichment analysis**

The relationships among the targets, expression, and related pathways of the microRNAs investigated in this study were visualized using the circlize and OmicCircos R packages. The outermost circle of the Circos plot indicated the binding sites of each miRNA on the chromosomes, which were identified using the mirWalk database version 3.0[15]. The heatmap in the Circos plot represented the expression levels of each miRNA across 13 different cancer types. Genes that were present in more than two-thirds of the databases (TargetScan, miRDB miRTarBase) were considered as meaningful targets and were selected for further evaluation [12-14]. To gain more insight regarding functions of these genes.

Gene ontology (GO) and Kyoto Encyclopedia of Genes and Genome (KEGG) analyses of genes regulated by these aforementioned miRNAs were performed using the Database for Annotation Visualization and Integrated Discovery (DAVID, https://david.ncifcrf.gov/, 15 October, 2022) [16]. A p-value < 0.05 and FDR < 0.05 were set as the cutoff criteria.

**The splitting strategy of dataset**

We implemented a standard pipeline for developing and evaluating a diagnostic classifier using machine learning algorithms. The available data were randomly divided into three independent subsets with different proportions: 70% of the randomly selected samples were used for training, 20% for validation, and the remaining 10% for testing. Additionally, an independent external test set was identified from the GSE113740 dataset, which included 13 cancer types and a control group with unique identities. The training and validation sets were used for classifier development, while the test set was used to assess the generalization and reproducibility of the signature. Detailed baseline information is summarized in **Table S1**.

**Differentially expressed miRNAs**

To identify reliable candidate cell-free immune-related miRNAs (cf-IRmiRNAs) that showed differential representation between malignant and non-malignant controls, we initially performed differential analysis on the entire training cohort. Differentially expressed miRNAs were identified using the limma package[17], with a cutoff value of |log-transformed fold change| >= 1 and adjusted *P*-value < 0.01. A total of 100 specific miRNAs were filtered as classification features. Subsequently, we applied Least Absolute Shrinkage and Selection Operator (LASSO) regression analysis to shrink the parameters and overcome multicollinearity. This method adds a penalty/regularization term to the cost function to further reduce model complexity and prevent overfitting. Based on the λ value, 39 miRNAs with beta coefficients above zero were retained in the model to calculate a discriminant score.

**Construction of diagnostic classifiers based on five machine-learning methods**

In order to construct a robust classifier capable of accurately and consistently discriminating between malignant and non-malignant samples, we employed five machine learning algorithms for binary classification to create predictive classification models. These five machine learning algorithms are Logistic regression, Lasso regression, Random Forest, Support Vector Machine (SVM), and XGBoost. Logistic regression is a generalized linear model (GLM) used to solve a wide range of binary classification problems. LASSO regression is a biased estimation method for regression that improves upon the least squares estimation method. It is particularly effective in handling multicollinearity, noisy, and redundant data, and can be used to analyze continuous dependent variables, binary, and multivariate discrete variables[18]. SVM is a machine learning algorithm used for classification and regression analysis. It finds the optimal hyperplane that can distinguish between two data classes by mapping the sample vectors to a high-dimensional space and maximizing the distance between each class and the hyperplane[19]. Random Forest uses multiple decision trees trained on different subsets of the same training data to reduce variance by averaging the predictions[20]. XGBoost, as a member of the boosting algorithm family, is highly scalable, especially for handling a large number of features and samples. This method uses a gradient boosting framework to build an ensemble of weak decision trees with the goal of minimizing the loss function by adjusting the weights of the samples and features[21].

According to the feature importance of each miRNA in different algorithms, 39 selected miRNAs were orderly enrolled for model construction in the training set. For each algorithm, we selected its optimal feature number by comparing the Youden index of models composed of 1-39 candidates in the validation set. The diagnostic performance of each algorithm’s best model was horizontally compared through the index of area under the receiver operating characteristic (ROC) curve (AUC).

**Hyperparameter tuning and optimization**

To overcome overfitting, we employed five-fold cross-validation to optimize the hyperparameters of the classifier in the training and validation sets. The samples were divided into five folds using random shuffling of the entire input dataset. In each iteration (looping from 1 to 5), the i^th^ fold was set as the validation set, and the remaining folds were used as the training set. The optimal hyperparameters of the classifiers were estimated by averaging the validation performance. The parameters is {'n_estimators': 70, 'max_depth': 6, 'min_child_weight': 1, 'gamma': 0.05, 'colsample_bytree': 1, 'subsample': 1, 'reg_alpha': 0, 'reg_lambda': 1, 'learning_rate': 0.3}.

**Validation and evaluation of the diagnostic performance of the classifier**

Then we applied the cf-IRmiRNAs signature in the test and external cohort to ensure its generalization ability. The ability of the classifier to distinguish between cancer with different types and non-cancer was also investigated by combining each cancer type individually with non-malignant samples. Considering the gender-specific incidence of certain cancer types, we only included female non-malignant controls when analyzing BRCA and OV and male non-malignant controls when analyzing PRAD. We also evaluated the diagnostic performance in early-stage cancers, including BLCA, COADREAD, ESCA, LIHC, LUNG, OV, PRAD, and SARC. To explore the potential application of the identified signature in differential diagnosis, we evaluated the performance of the model in distinguishing cancer from benign lesions of different organs or tissues, including soft tissues, breast, liver, ovary, and prostate.

**Statistical analysis**

Basic information about the clinical cohort and the general situation of the patients were presented by frequency (proportion) for categorical data and mean (standard deviation, SD) or median (interquartile range, IQR) for quantitative data. The individuals were randomly divided into training, validation, and test sets, and the training and validation sets were used for classifier construction and fitting. Final trained models were finalized with fixed weights on test and external test sets. The predictive performance and generalization ability of cancer detection were evaluated on test and external test sets. Evaluation indicators include sensitivity, specificity, positive predictive value (PPV), negative predictive value (NPV), accuracy, the AUC and Precision-Recall (P-R) Curve. The confounding matrix was used to summarize the prediction results of the classifier and recorded according to the standards of “real category” and “predicted category”. TP, FP, FN, and TN represent true positive, false positive, false negative, and true negative, respectively. ROC analysis was mainly used to estimate the diagnostic performance of the identified signature. It was constructed with (1-specificity) as abscissa and sensitivity as ordinate. The value of AUC is between 0.5-1, and a larger value indicates better performance. P-R curve is a graph with precision on the y-axis and recalls on the x-axis for different probability thresholds.

We mainly used packages of “numpy”, “pandas”, “xgboost”, “sklearn.linear_model.LassoCV”, “sklearn.linear_model. LogisticRegression”, “sklearn.ensemble.RandomForestClassifier”, and “sklearn.svm” in Python to construct the diagnostic classifiers. All data processing, statistical analysis, and plotting were conducted using R 3.4.3 (R Development Core Team; <http://www.r-project.org>) and Python 3.10; Python Software Foundation, <https://www.python.org/downloads/release/> python-3100/). The significance level (α) was set at 0.05, and all tests were two-sided.


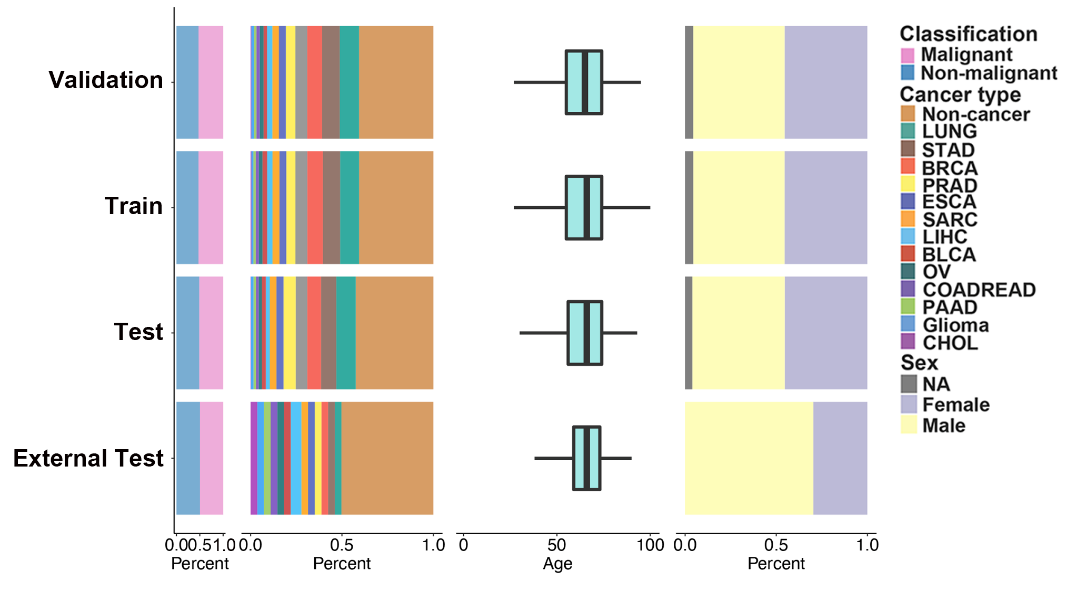


**Fig S1.** Distribution of the number of samples, histological type, age, and sex in partition datasets.


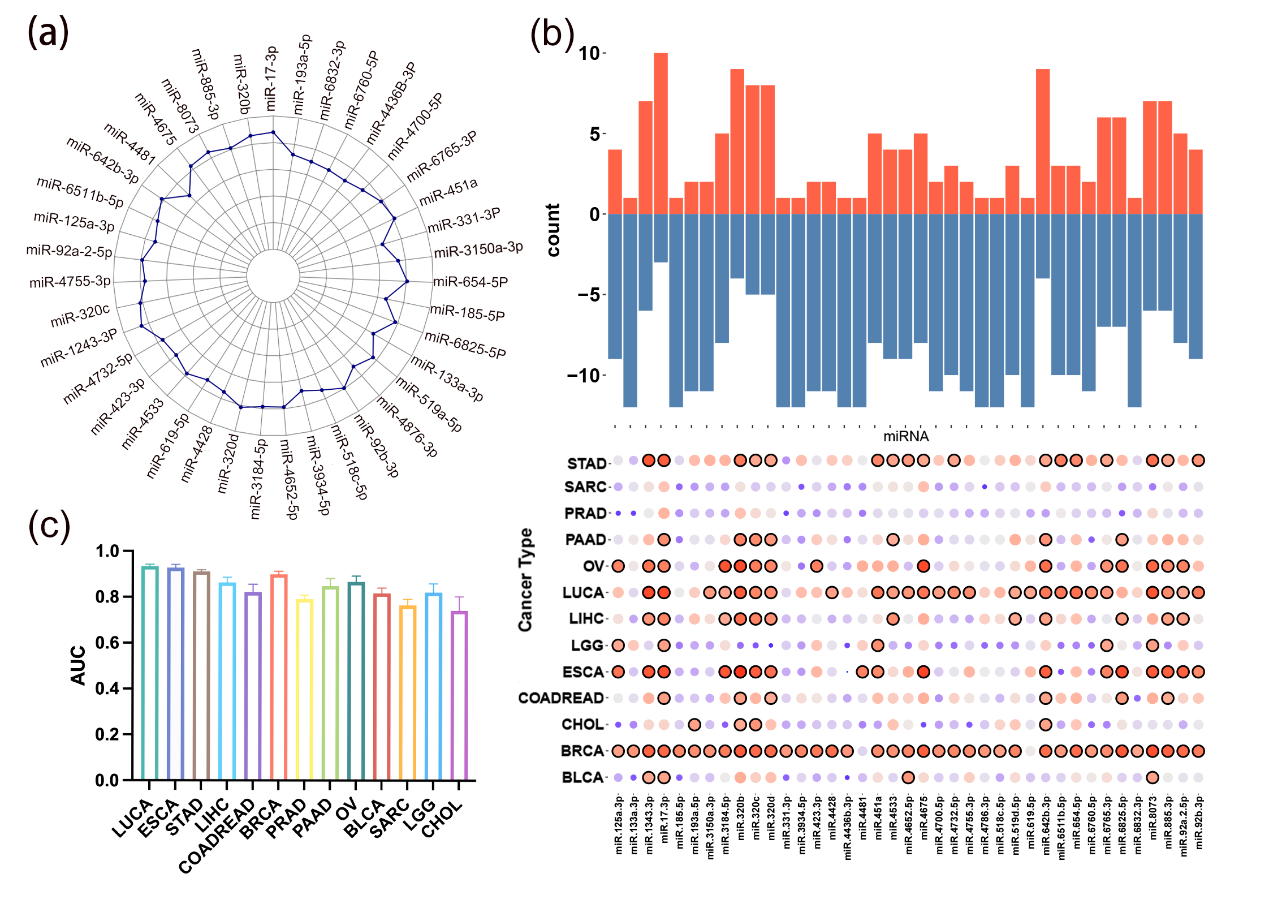


**Fig S2.** The performance of each candidate miRNA in training set. a. The radar chart summarized the area under the curve (AUC) of each miRNA. b. The diagnostic utility of has-miR-17-3p in distinguishing malignancies and non-malignancies. c. Bar plot of the AUC estimation in different cancer types.


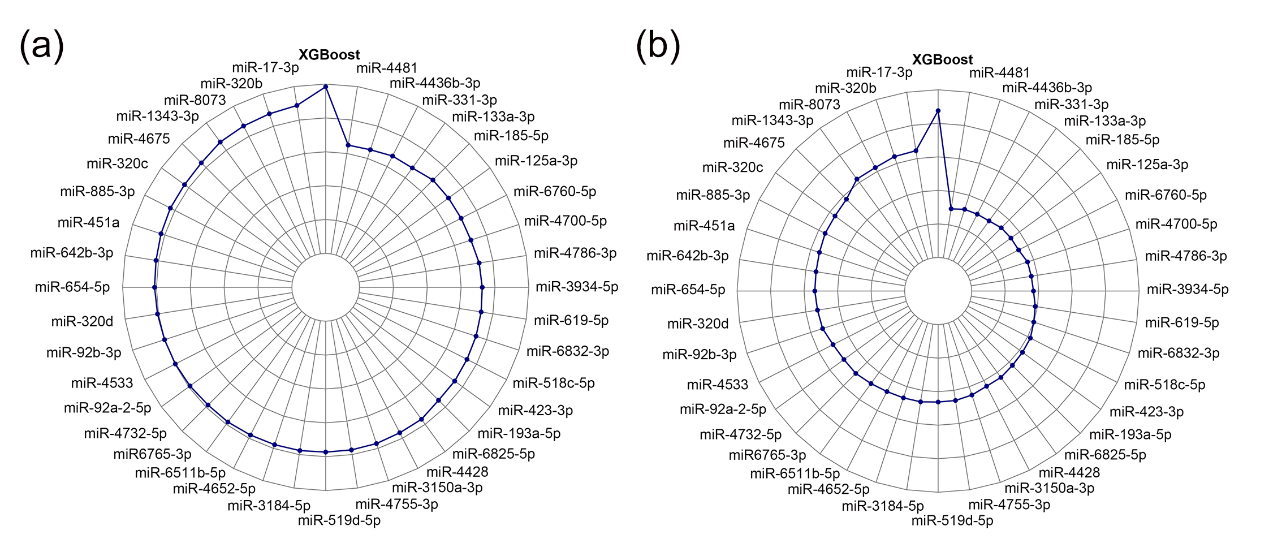


**Fig S3.** Comparison of diagnostic efficacy between single miRNA and model. a-b. Radar chart summarized the Youden index and area under the curve (AUC) of the single and XGBoost models.


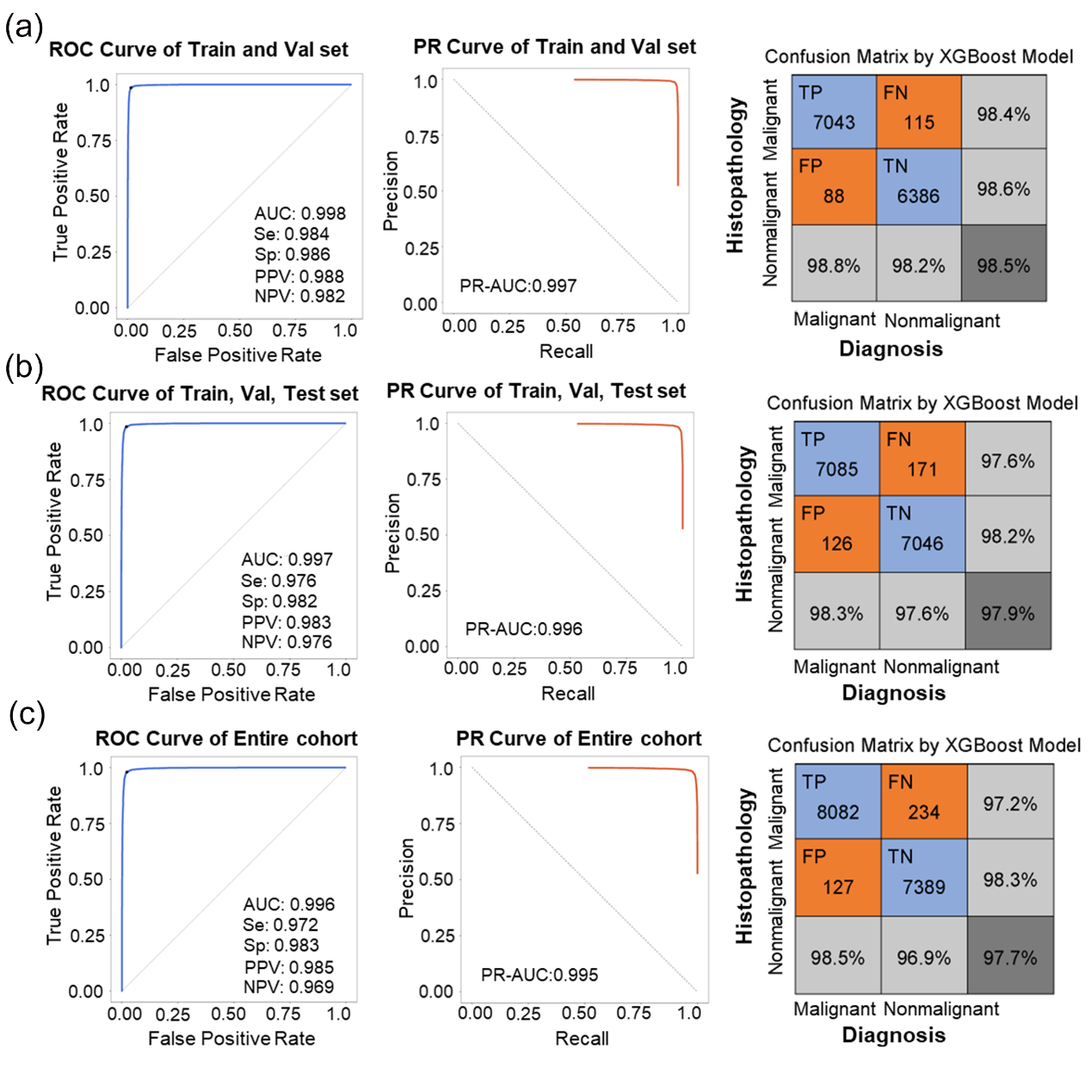


**Fig S4.** Validation of cell free immune-related miRNA (cf-IRmiRNAs) signature in clinical cohort. a. ROC, PR curve, and confusion matrix for cf-IRmiRNAs signature for cancer diagnosis in a. the data set consisting of training and validation sets. b. the data set consisting of training, validation, and test sets. c. Entire cohort.


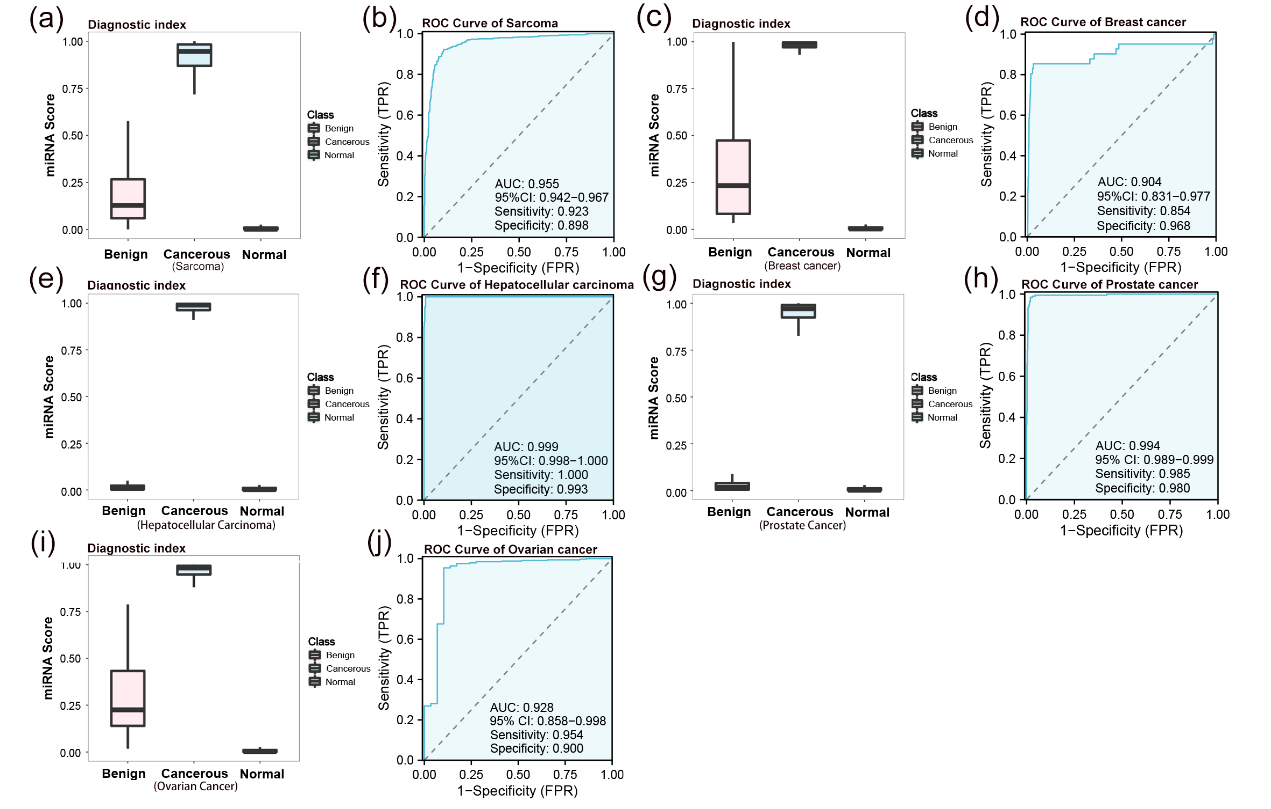


**Fig S5.**  The utility of the diagnostic index in discriminating benign diseases and malignant tumors. a. The diagnostic index of benign bone and soft tissue tumors or diseases, sarcoma, and normal samples. b. Roc curves showed the discriminant ability in benign bone and soft tissue tumors or diseases and sarcoma. c. The diagnostic index of benign breast disease, breast cancer, and normal samples. d. Roc curves showed the discriminant ability in benign breast disease and breast cancer. e. The diagnostic index of benign liver disease (chronic hepatitis or liver cirrhosis), hepatocellular carcinoma, and normal samples. f. Roc curves showed the discriminant ability in benign liver disease (chronic hepatitis or liver cirrhosis) and hepatocellular carcinoma. g. The diagnostic index of benign prostate disease, prostate cancer, and normal samples. h. Roc curves showed the discriminant ability in benign prostate disease and prostate cancer. i. The diagnostic index of benign ovarian disease, ovarian cancer, and normal samples. j. Roc curves showed the discriminant ability in benign ovarian disease and ovarian cancer.

**References**

1. Asakura K, Kadota T, Matsuzaki J, Yoshida Y, Yamamoto Y, Nakagawa K, Takizawa S, Aoki Y, Nakamura E, Miura J, et al. A mirna-based diagnostic model predicts resectable lung cancer in humans with high accuracy. Commun Biol. 2020;3(1):134. https://doi.org/10.1038/s42003-020-0863-y.

2. Sudo K, Kato K, Matsuzaki J, Boku N, Abe S, Saito Y, Daiko H, Takizawa S, Aoki Y, Sakamoto H, et al. Development and validation of an esophageal squamous cell carcinoma detection model by large-scale microrna profiling. JAMA Netw Open. 2019;2(5):e194573. https://doi.org/10.1001/jamanetworkopen.2019.4573.

3. Asano N, Matsuzaki J, Ichikawa M, Kawauchi J, Takizawa S, Aoki Y, Sakamoto H, Yoshida A, Kobayashi E, Tanzawa Y, et al. A serum microrna classifier for the diagnosis of sarcomas of various histological subtypes. Nat Commun. 2019;10(1):1299. https://doi.org/10.1038/s41467-019-09143-8.

4. Usuba W, Urabe F, Yamamoto Y, Matsuzaki J, Sasaki H, Ichikawa M, Takizawa S, Aoki Y, Niida S, Kato K, et al. Circulating mirna panels for specific and early detection in bladder cancer. Cancer Sci. 2019;110(1):408-19. https://doi.org/10.1111/cas.13856.

5. Yokoi A, Matsuzaki J, Yamamoto Y, Yoneoka Y, Takahashi K, Shimizu H, Uehara T, Ishikawa M, Ikeda SI, Sonoda T, et al. Integrated extracellular microrna profiling for ovarian cancer screening. Nat Commun. 2018;9(1):4319. https://doi.org/10.1038/s41467-018-06434-4.

6. Zhang A, Hu H. A novel blood-based microrna diagnostic model with high accuracy for multi-cancer early detection. Cancers (Basel). 2022;14(6). https://doi.org/10.3390/cancers14061450.

7. Yamamoto Y, Kondo S, Matsuzaki J, Esaki M, Okusaka T, Shimada K, Murakami Y, Enomoto M, Tamori A, Kato K, et al. Highly sensitive circulating microrna panel for accurate detection of hepatocellular carcinoma in patients with liver disease. Hepatol Commun. 2020;4(2):284-97. https://doi.org/10.1002/hep4.1451.

8. Urabe F, Matsuzaki J, Yamamoto Y, Kimura T, Hara T, Ichikawa M, Takizawa S, Aoki Y, Niida S, Sakamoto H, et al. Large-scale circulating microrna profiling for the liquid biopsy of prostate cancer. Clin Cancer Res. 2019;25(10):3016-25. https://doi.org/10.1158/1078-0432.CCR-18-2849.

9. Ohno M, Matsuzaki J, Kawauchi J, Aoki Y, Miura J, Takizawa S, Kato K, Sakamoto H, Matsushita Y, Takahashi M, et al. Assessment of the diagnostic utility of serum microrna classification in patients with diffuse glioma. JAMA Netw Open. 2019;2(12):e1916953. https://doi.org/10.1001/jamanetworkopen.2019.16953.

10. Shimomura A, Shiino S, Kawauchi J, Takizawa S, Sakamoto H, Matsuzaki J, Ono M, Takeshita F, Niida S, Shimizu C, et al. Novel combination of serum microrna for detecting breast cancer in the early stage. Cancer Sci. 2016;107(3):326-34. https://doi.org/10.1111/cas.12880.

11. Gene ontology consortium: going forward. Nucleic Acids Res. 2015;43(Database issue):D1049-56. https://doi.org/10.1093/nar/gku1179.

12. Lewis BP, Shih IH, Jones-Rhoades MW, Bartel DP, Burge CB. Prediction of mammalian microrna targets. Cell. 2003;115(7):787-98. https://doi.org/10.1016/s0092-8674(03)01018-3.

13. Huang HY, Lin YC, Cui S, Huang Y, Tang Y, Xu J, Bao J, Li Y, Wen J, Zuo H, et al. Mirtarbase update 2022: an informative resource for experimentally validated mirna-target interactions. Nucleic Acids Res. 2022;50(D1):D222-30. https://doi.org/10.1093/nar/gkab1079.

14. Chen Y, Wang X. Mirdb: an online database for prediction of functional microrna targets. Nucleic Acids Res. 2020;48(D1):D127-31. https://doi.org/10.1093/nar/gkz757.

15. Dweep H, Sticht C, Pandey P, Gretz N. Mirwalk--database: prediction of possible mirna binding sites by "walking" the genes of three genomes. J Biomed Inform. 2011;44(5):839-47. https://doi.org/10.1016/j.jbi.2011.05.002.

16. Huang DW, Sherman BT, Lempicki RA. Systematic and integrative analysis of large gene lists using david bioinformatics resources. Nat Protoc. 2009;4(1):44-57. https://doi.org/10.1038/nprot.2008.211.

17. Ritchie ME, Phipson B, Wu D, Hu Y, Law CW, Shi W, Smyth GK. Limma powers differential expression analyses for rna-sequencing and microarray studies. Nucleic Acids Res. 2015;43(7):e47. https://doi.org/10.1093/nar/gkv007.

18. Tibshirani R. Regression shrinkage and selection via the lasso. Journal of the Royal Statistical Society Series B: Statistical Methodology. 1996;58(1):267-88.

19. Boser BE, Guyon IM, Vapnik VN. A training algorithm for optimal margin classifiers. 1992;144-52. https://doi.org/.

20. Breiman L. Random forests. Mach Learn. 2001;45(5-32.

21. Chen T, Guestrin C. Proceedings of the 22nd acm sigkdd international conference on knowledge discovery and data mining. 2016.
